# A possible role for immunogenetic factors in myositis developing after vaccination in the pre-covid-19 era

**DOI:** 10.3389/fimmu.2025.1539659

**Published:** 2025-08-15

**Authors:** Eaman Alhassan, Anna Patnaik, Ejaz A. Shamim, Janardan P. Pandey, Lisa G. Rider, Frederick W. Miller

**Affiliations:** ^1^ Division of Rheumatology and Clinical Immunology, Department of Medicine, University of Pittsburgh Medical Center, Pittsburgh, PA, United States; ^2^ Department of Neurology, Johns Hopkins University, Baltimore, MD, United States; ^3^ Environmental Autoimmunity Group, Clinical Research Branch, National Institute of Environmental Health Sciences, National Institutes of Health, Bethesda, MD, United States; ^4^ Mid-Atlantic Permanente Medical Group and Mid-Atlantic Permanente Research Institute, Washington DC, United States; ^5^ National Institutes of Health, National Institute of Neurological Disorders and Stroke, Bethesda, MD, United States; ^6^ Department of Pharmacology and Immunology, Medical University of South Carolina, Charleston, SC, United States; ^7^ Environmental Autoimmunity Group, Clinical Research Branch, National Institute of Environmental Health Sciences, National Institutes of Health, Durham, NC, United States

**Keywords:** polymyositis, dermatomyositis, vaccination, adverse events, HLA, GM/KM, juvenile dermatomyositis

## Abstract

**Introduction:**

Vaccinations have had a transformative impact on public health, reducing the incidence of many infectious diseases and increasing survival. However, there remains uncertainty about the potential of vaccines to trigger autoimmune diseases such as the idiopathic inflammatory myopathies (IIM). Myositis after vaccination (MAV) is a rare clinical entity, but given immunogenetic associations with other adverse events, we explored genetic risk factors, particularly human leukocyte antigen (HLA) alleles and GM/KM immunoglobulin allotypes, that may predispose individuals to develop MAV.

**Methods:**

We examined clinical characteristics, vaccination history, autoantibodies, HLA alleles and GM/KM allotypes from 56 patients who developed MAV, 133 myositis cases with no documented vaccination within 6 months of onset (non-MAV), and 527 healthy controls from the pre-COVID-19 era. Genotyping for HLA and GM/KM allotypes was performed by standard assays. Differences in allele frequencies in race-matched groups were evaluated using chi-square tests, odds ratios (OR) and 95% confidence intervals (CI). Multivariate logistic regression adjusted for age, sex, and vaccination type. Statistical significance was defined as a Holms corrected p-value of less than 0.05.

**Results:**

No clinical or serologic differences were found between MAV and non-MAV patients. However, the HLA-DQA1*03:03 allele was a unique risk factor for MAV in Caucasians (OR=3.87, 95% CI=1.56-9.54, p=0.002), while the known myositis risk factor, HLA-DRB1*03:01, was a protective factor for MAV (OR=0.41, 95% CI=0,18-0.94, p= 0.033). GM2, GM13, and KM1 allotypes were more frequently observed in MAV patients than healthy controls, and other HLA alleles were risk or protective factors for specific vaccines given in patients who developed MAV.

**Conclusion:**

Immunogenetic factors may influence the likelihood of developing MAV. Further studies of larger, deeply phenotyped populations are needed to confirm these associations and could inform personalized risk assessments and targeted interventions, thereby enhancing vaccine safety.

## 1 Introduction

The idiopathic inflammatory myopathies (IIM) are a group of rare systemic autoimmune conditions characterized by muscle inflammation and weakness that arise from chronic immune activation in genetically predisposed individuals in response to certain environmental triggers (1). Major strides have been made in defining the genetic risks for IIM and other autoimmune conditions (2), but identifying the even more important environmental risk factors has been hampered by the lack of validated measures and the constantly changing mixtures of exposures that occur over a lifetime (3). Vaccines, while highly beneficial, can in rare cases, cause chronic immune activation followed by the development of a number of autoimmune diseases, including myositis (4, 5).

Certain polymorphic immune response genes have been associated with IIM. One of the strongest genetic associations for autoimmune diseases is located on chromosome 6p21.3 that includes the human leukocyte antigen (HLA) locus in addition to other immune system-modulating genes (6). Alleles of the 8.1 ancestral haplotype (8.1 AH), HLA-DRB1*03:01 and HLA-B*08:01, show the strongest association with IIM in Caucasians (7, 8). Other polymorphic genes associated with autoimmune diseases, including IIM, are the immune response genes that encode immunoglobulin gamma heavy chains (GM) and immunoglobulin kappa light chains (KM) (9). These have also been identified as genetic susceptibility factors across different ages and ethnicities for various clinical and serological IIM phenotypes (10).

There is no doubt that vaccines have significantly improved global public health by boosting immune responses to many infectious agents, preventing infections, and minimizing morbidity and mortality. However, it is plausible that vaccines, often given intramuscularly, could cause initial immune activation in muscles to progress to a chronic systemic inflammatory response in those with certain immunogenetic backgrounds. While many patients develop myositis without any documented recent vaccination, the concept that vaccinations may be linked to the onset of some cases of myositis has been previously suggested in case reports (11–14). The first identified cases of myositis following vaccination (MAV) included myositis developing in a temporally related way to diphtheria-tetanus-pertussis vaccines (11, 15, 16) and smallpox vaccines (17) in adult and pediatric patients. Additional reports of vaccine constituents, including aluminum hydroxide, and not the immunization antigens themselves, have led to macrophagic myofasciitis (18).

Certain adverse events to drugs, medical implants and vaccines have previously been associated with clinical, serologic or immunogenetic features (19–21). Based on our observation that some myositis cases were temporally associated with vaccinations, we systematically compared those patients who developed myositis within 6 months of a documented vaccination to those who had no documented vaccinations within 6 months of myositis disease onset and to healthy controls (HC) from the pre-COVID-19 era to assess possible clinical, serological, and immunogenetic differences.

## 2 Materials and methods

### 2.1 Study participants

Myositis patients and HC were enrolled into investigational review board-approved clinical protocols at the National Institutes of Health (NIH) Warren Grant Magnuson Clinical Center and the United States Food and Drug Administration from 1983 to 2002. These protocols studied the natural history of myositis and twins and siblings discordant for myositis.

Per our protocol criteria, all patients met Bohan and Peter criteria for definite or probable myositis (22, 23). They were all diagnosed with IIM, including dermatomyositis (DM), juvenile dermatomyositis (JDM), polymyositis (PM), juvenile polymyositis (JPM), and inclusion body myositis (IBM) based on the accepted criteria at the time of enrollment. Patients with myositis and another connective tissue disease (CTM) were also included. IIM patients who received a documented vaccination within six months prior to first myositis symptom onset were included in the myositis after vaccination (MAV) group (n=56), while those who did not receive vaccination during this time interval (documented by history and review of medical records) were categorized as non-MAV (n=133). All patients underwent a comprehensive medical history and physical examination, which included detailed protocol questionnaires completed by the patients and their enrolling physicians.

The clinical data included age, self-classified race, gender, and signs and symptoms. Since gene frequencies differ by race, the HLA and GM/KM data were assessed in Caucasian patients, which was the largest cohort and the only one adequate for reliable statistical analysis. The HC groups were race-matched.

### 2.2 HLA typing

HLA allele typing was performed using purified genomic DNA, using laboratory-designed and commercial reagents (Genovision, West Chester, PA; Dynal Biotech, Lafayette Hill, PA) and PCR-mediated sequence-specific oligonucleotide probe hybridization and sequence-specific priming technique via standard techniques (24).

Allele frequencies per patient (carriage rates) were determined by the number of allele-positive subjects divided by the total number of subjects for which complete HLA data were available at a given locus. All patients in the HLA allele analysis were self-identified as Caucasians and divided into MAV (n=48) and non-MAV (n=93) groups. For comparison, the HC data (n=527), who did not have myositis, were obtained through the NIH HLA laboratory.

### 2.3 GM and KM allotyping

Immunoglobulin gamma heavy chain (GM) and immunoglobulin kappa light chain (KM) allotyping was performed using standard hemagglutination inhibition methods to type for IgG1m, IgG2m, and IgG3m and for IgKM1 and IgKM3 (25). Allotype and phenotype frequencies were determined by the number of allotype-positive subjects divided by the total number of subjects for which data were available at a given locus. All patients in the GM and KM allotype analysis were Caucasian and divided into MAV (n=19) and non-MAV (n=34) cases. Race-matched HC (n=266) were used for comparison.

### 2.4 Autoantibody identification

Myositis-specific autoantibodies (anti-synthetases, anti-signal recognition particle (anti-SRP), anti–Mi-2 and myositis-associated autoantibodies (anti-Ku, anti-La, anti-Ro, anti–URNP, and anti–PM-Scl), were identified from frozen serum samples using previously validated methods of protein and RNA immunoprecipitation (IPP) and double immunodiffusion (10). The NXP2 and TIF1 autoantibodies were identified with IPP, followed by immunoblotting (26).

### 2.5 Statistical analysis

Analyses were performed using GraphPad Prism (GraphPad, Inc., La Jolla, CA). For both the HLA allele analysis and the GM/KM allotype analysis, the allele or allotype frequencies were compared by chi-square test or Fisher’s exact test for counts below 5, for 2x2 contingency tables between MAV and controls, MAV and non-MAV, or non-MAV and controls. The odds ratios (OR), 95% confidence intervals (CI) were determined. The MAV group was also divided and compared to non-MAV and HC by the four most frequent vaccines: Hepatitis B, Influenza, Tetanus, and Mumps-Measles-Rubella (MMR).

A p-value was considered significant if below 0.05 using the Holm procedure to adjust for multiple comparisons (27). The U-test, or Mann-Whitney test, was used to compare non-parametric variables, such as the months from vaccine to first symptom, calculations between children and adults, and between the different vaccines.

Chi-square tests were performed to examine differences in the frequency distributions between the MAV and non-MAV groups. An analysis in which the distribution of clinical subgroups significantly differed between the MAV and non-MAV groups led to performing a sensitivity analysis, in which a random sample of patients were selected in similar clinical subgroups. This was also performed with the MAV group within 6 months and 3 months from vaccination. If the genetic results differed from the primary analysis, the difference in clinical subgroup distribution was interpreted to have affected the result, however, if the genetic results remained the same, the difference in clinical subgroup distribution was interpreted as not affecting the genetic results.

## 3 Results

### 3.1 Clinical findings

There were 56 patients, including 28 females, in the MAV group, 48 of whom were Caucasian, three African American, and five of mixed race, and 133 patients, including 92 females, in the non-MAV group, of which 98 were Caucasian, 12 African American, six Asian or Hispanic, and 17 of mixed race. Of these, 48 MAV patients and 95 non-MAV patients were Caucasian and HLA-typed, while 19 MAV patients and 34 non-MAV patients were Caucasian and also underwent GM/KM typing. The clinical and autoantibody subgroup, race, gender, and signs and symptom distributions were similar in the MAV and non-MAV groups for all patients included in the study (**Table 1**), as well as for the HLA-analyzed groups. The patients in which GM/KM was examined had a lower frequency of JDM in the MAV group (21.1%) and a higher frequency of JDM in the non-MAV group (73.5%) (p = 0.0004). The median age of disease onset for the MAV group was 5.4 years in children and 43.8 years in adults, which was similar to the non-MAV group (6.7 and 45.4 years, respectively).

**Table 1:** Distribution of clinical and autoantibody subgroups, and signs and symptoms of myositis patients developing symptoms of myositis within 6 months of vaccination (MAV) and those without documented vaccination within 6 months of symptom onset (non-MAV).

Clinical Groups*	MAV (n=56)	Non-MAV (n=133)
	N (%)	N (%)
JDM	26 (46.4)	84 (63.2)
DM	10 (17.8)	17 (12.8)
PM	13 (23.2)	15 (11.3)
CTM	3 (5.4)	7 (5.3)
IBM	2 (3.6)	6 (4.5)
JPM	2 (3.6)	4 (3.0)
Myositis-Autoantibody Groups*+		
MSA and MAA Negative	36 (64.3)	89 (66.9)
p155 (TIF1)	14 (25.0)	40 (30.1)
Mi-2	5 (8.9)	6 (4.5)
MJ (NXP2)	3 (5.4)	23 (17.3)
SRP	3 (5.4)	6 (4.5)
Aminoacyl tRNA-Synthetases	4 (7.2)	11 (8.3)
Ro60	5 (8.9)	12 (9.0)
PM-Scl	2 (3.6)	4 (3.0)
U1RNP	1 (1.8)	6 (4.5)
Clinical Features*		
Myalgia	35 (63.6)	88 (67.2)
Distal muscle weakness	29 (51.8)	62 (47.0)
Muscle atrophy	27 (50.0)	51 (38.6)
Falling	25 (46.3)	62 (47.3)
Dysphagia	24 (44.4)	61 (46.2)
Cuticular overgrowth	22 (40.7)	41 (31.3)
Fever	20 (35.7)	42 (31.8)
Arthritis	17 (30.4)	58 (43.9)
V-sign rash	17 (31.5)	38 (29.0)
Asymmetric weakness	12 (22.2)	21 (16.2)
Raynaud’s Phenomenon	12 (22.2)	18 (13.6)
Shawl-sign rash	9 (16.7)	25 (19.1)
Mechanic’s hands	7 (13.0)	13 (9.9)
Palpitations	4 (7.4)	12 (9.2)
Carpal Tunnel Syndrome	4 (7.4)	7 (5.3)
Interstitial lung disease	2 (3.8)	8 (6.1)

#MAV, myositis symptoms developing within 6 months of documented vaccination; non-MAV, no documented immunization within 6 months of onset of myositis; JDM, juvenile dermatomyositis; DM, dermatomyositis; PM, polymyositis; CTM, connective tissue disease overlap with myositis; IBM, inclusion body myositis; JPM, juvenile polymyositis; MSA, myositis-specific autoantibody; MAA, myositis-associated autoantibody; p155 (TIF1), anti-transcription intermediary factor 1 autoantibodies; MJ (NXP2), anti-nuclear matrix protein autoantibodies 2; SRP, anti-signal recognition particle autoantibodies; Ro60, autoantibodies to the 60kD protein of the heterogeneous antigenic complex; PM-Scl, autoantibodies to the 75kD and 100kD proteins seen in the polymyositis/scleroderma complex; U1RNP, autoantibodies to the U1 ribonucleoprotein complex.

+ Sum is > 100%, as some patients have both MSA and MAA.

*No significant differences were detected between the MAV and Non-MAV groups.

Of the 56 MAV patients, 17 received a form of the tetanus vaccine, 15 received a Hepatitis B vaccine, 15 received an influenza vaccine, and 13 received a MMR vaccine (**Table 2**). The median time to myositis symptoms after vaccination was 2.2 months with a range of 0–6 months and an IQR of 3.5 months, while the median time to diagnosis of myositis after vaccination was 7.0 months. Tetanus, influenza, and MMR had a similar period from vaccination to first myositis symptom. However, for those who received Hepatitis B vaccine, there was a significantly shorter latency period, with a median of 1 month from vaccination to first myositis symptom (p = 0.045). In the cases where vaccines were given in a series, there was a median of 2.2 months from the time of first vaccination to first myositis symptom, a median of 3.0 months after the second vaccine, and a median of 3.5 months after the third vaccine.

**Table 2:** Distribution of the number of patients receiving vaccines and the number of vaccines administered prior to first symptoms in 56 patients who developed myositis within 6 months after vaccination#.

Vaccine	Patients receiving a vaccine within 6 months of onset (% of all 56 patients)+	Vaccinations administered within 6 months of onset (% of all 98 vaccinations)
Any Tetanus (DPT, DTaP, or Td)	17 (30.4)	18 (18.4)
Hepatitis B	15 (26.8)	27 (27.6)
Influenza A/B	15 (26.8)	15 (15.3)
MMR or Measles	13 (23.2)	13 (13.3)
OPV or IPV	6 (10.7)	6 (6.1)
Prevnar or Pneumococcal	3 (5.4)	3 (3.1)
Hepatitis A, Hemophilus influenzae type B, Varicella, Meningococcal, Typhoid, or Yellow fever *	12 (21.4)	12 (12.2)
Rabies, Japanese Encephalitis, Influenza A virus subtype H1N1, or Lyme **	4 (7.2)	4 (4.0)

*2 patients each received one of these 6 vaccines, and 2 vaccinations were administered for each vaccine listed.

**1 patient each received one of these 4 vaccines, and 1 vaccination was administered for each vaccine listed.

DPT, diphtheria pertussis tetanus vaccine; DTaP, diphtheria tetanus acellular pertussis vaccine; Td, tetanus booster; MMR, measles mumps rubella vaccine; OPV, oral polio vaccine; IPV, inactivated polio vaccine.

+Ten patients received more than 1 vaccine at the same time and the combinations of vaccines given within 6 months of developing myositis were: Patient 1 - 1st HepB, influenza; Patient 2 - 1st HepB, MMR; Patient 3 – OPV, MMR; Patient 4 - DTP, Haemophilus influenzae type B, 3rd HepB, pneumococcal conjugate vaccine; Patient 5 – DTaP, HIB, 3rd HepB; Patient 6 – DTaP, IPV, MMR; Patient 7 – DTaP, OPV; Patient 8 – Td, MMR; Patient 9 – Varicella, MMR; and Patient 10 – Td, HepA.

In total, 98 vaccines were administered to the 56 patients (**Table 2**). Sixteen patients received multiple vaccines on different days within the 6-month period and nine patients received 2 or 3 doses of Hepatitis B vaccine. Among 16 Hepatitis B patients, five developed MAV after the 1^st^ dose, five developed MAV after the 2^nd^dose, and six developed MAV after the 3^rd^ dose.

### 3.2 HLA analysis

The frequency of DQA1*02:01 was significantly higher in the MAV group compared to non-MAV (OR = 3.80, 95% CI = 1.36-10.58, p = 0.007), however, it was protective for non-MAV versus HC (OR = 0.25, 95% CI = 0.11-0.55, p = 0.0004) (**Table 3**). The frequency of DRB1*03:01 was significantly lower for MAV compared to non-MAV (OR = 0.41, 95% CI = 0.18-0.94, p = 0.033) but it was a risk factor for the non-MAV versus HC (OR = 3.42, 95% CI = 2.14-5.48, p < 0.0001), but not for MAV vs. HC. DRB1*15 was a protective factor for the non-MAV group compared to HC (OR = 0.44, 95% CI = 0.22-0.88, p = 0.017). DQA1*05 was a risk factor for the non-MAV group (OR = 2.25, 95% CI = 1.40-3.45, p = 0.004). Adult and juvenile data were similar in the overall HLA analysis and showed no significant differences.

**Table 3:** Differences in HLA types in Caucasian myositis after vaccination (MAV) patients, non-MAV patients, and healthy controls*.

HLA Alleles	MAV % (n=48)	Non-MAV % (n=93)	Control % (n=527)	MAV vs. Non-MAV		MAV vs. Healthy Control		Non-MAV vs. Healthy Control	
				P-value	OR (95% CI)	P-value	OR (95% CI)	P-value	OR (95% CI)
HLA-DRB1									
*02	0.0	2.2	26.8	0.543	1.05 (0.09-11.93)	<0.0001	0.03 (0.01-0.46)	<0.0008	0.06 (0.01-0.25)
*03:01	26.3	45.6	20.3	0.033	0.41 (0.18-0.94)	0.498	0.71 (0.33-1.51)	<0.0001	3.42 (2.14-5.48)
*07:01	26.3	12.5	23.9	0.099	0.40 (0.15-1.04)	0.888	0.88 (0.42-1.86)	0.173	0.45 (0.23-0.88)
*10:01	7.9	5.7	0.9	0.697	0.70 (0.16-3.10)	0.0125	8.95 (2.05-39.0)	0.0012	6.29 (1.78-22.20)
*15	12.5	10.8	21.4	0.976	0.84 (0.28-2.47)	0.202	1.91 (0.79-4.61)	0.0172	0.44 (0.22-0.88)
HLA-DQA1									
*02:01	23.4	7.4	24.3	0.007	3.80 (1.36-10.58)	0.920	1.04 (0.51-2.11)	0.0004	0.25 (0.11-0.55)
*03:01	19.1	22.3	7.7	0.823	1.21 (0.51-2.91)	0.007	4.23 (1.92-9.32)	<0.0001	3.43 (1.92-6.13)
*03:03	14.9	7.4	4.3	0.231	0.46 (0.15-1.40)	0.002	3.86 (1.56-9.54)	0.293	0.56 (0.23-1.35)
*05	47.9	62.1	42.6	0.105	1.78 (0.88-3.59)	0.544	0.81 (0.45-1.46)	0.004	2.2 (1.40-3.45)

*Carriage rates were determined by the number of allele-positive subjects divided by the number of subjects for whom complete HLA data were available at a given locus. Abbreviations per prior tables.

Several risk and protective alleles for the non-MAV group were also shared by the MAV group, including DRB1*10:01 (OR = 6.29, 95% CI = 1.78-22.20, p = 0.001) and DQA1*03:01 (OR = 3.43, 95% CI = 1.92-6.13, p < 0.0001) as risk factors. DRB1*02 (OR = 0.06, 95% CI = 0.01-0.25, p < 0.0008) was a protective factor for the non-MAV and MAV groups (**Table 3**). Homozygosity of HLA alleles did not show a significant impact for either risk or protective factors for the MAV or non-MAV groups.

Several HLA alleles demonstrated significant associations in the MAV versus HC groups (**Table 3**). The DRB1*10:01 allele was significantly associated with MAV (OR = 8.95, 95% CI = 2.05-39.00, p = 0.012) compared to HC. The DQA1 03:01 allele (OR = 4.23, 95% CI = 1.92-9.32, p = 0.007) and DQA1*03:03 (OR = 3.86, 95% CI = 1.56-9.54, p = 0.002) were also risk factors for MAV when compared to HC. HLA DQA1*03:03 was the only unique risk factor allele for MAV that was not also a risk for the non-MAV group when compared to HC (**Table 3**). However, the frequency of DRB1*02 (OR = 0.03, 95% CI = 0.01-0.46, p < 0.0001) was lower in MAV, indicating a lower likelihood of MAV in individuals with this allele. A sensitivity analysis of HLA alleles of MAV cases developing within three months of vaccination resulted in the same findings.

The frequencies of the linked alleles DQA1*02:01 and DRB1*07:01 were significantly higher in the MAV group receiving the Hepatitis B or influenza vaccines compared to non-MAV (**Table 4**). The DQA1*03:03 allele was a risk factor for MAV patients who received influenza vaccines compared to HC (**Table 4**).

**Table 4:** Differences in HLA types in Caucasian myositis after vaccination (MAV) patients, non-MAV patients, and healthy controls by vaccine types*.

Vaccine	HLA Alleles	MAV vs. Non-MAV		MAV vs. Control	
		P-value	OR (95% CI)	P-value	OR (95% CI)
Hepatitis B (n=7)	DRB1*07:01	0.006	14.00 (2.84-76.39)	0.018	7.92 (1.81-41.83)
	DQA1*02:01	0.002	16.57 (3.63-71.83)	0.037	5.23 (1.38-20.89)
Influenza (n=14)	DQA1*01	0.038	0.28 (0.01-0.93)	0.021	0.27 (0.10-0.79)
	DQA1*02:01	0.001	7.77 (2.05-26.21)	0.323	1.96 (0.71-6.26)
	DQA1*03:01	0.497	1.54 (0.20-2.06)	0.017	5.30 (1.73-17.83))
	DQA1*03:03	0.102	3.72 (0.92-14.26)	0.020	6.61 (1.84-25.68))
Tetanus (n=10)	DRB1*16	0.030	7.46 (1.64-36.91)	0.011	8.98 (2.38-35.76)

*Carriage rates were determined by the number of allele-positive subjects divided by the number of subjects for whom complete HLA data were available at a given locus; MAV patients in each group were compared to 93 non-MAV and 527 controls.

### 3.3 GM/KM analysis

The GM phenotype 1, 2, 3, 5, 13, 17, 21, 23 and allotypes GM 2 (OR = 3.17, 95% CI = 1.24-8.13, p = 0.012) and GM13 (OR = 12.5, 95% CI = 1.64-95.05, p = 0.001) were risk factors for MAV compared to HC, but were not risk factors for the non-MAV group (**Table 5**). KM1 (OR = 3.43, 95% CI = 1.30-9.03, p = 0.009), and KM1,3 (OR = 5.19, 95% CI = 1.47-18.29, p = 0.008) were also risk factors for MAV.

**Table 5:** Differences in GM/KM allotypes and phenotypes in Caucasian myositis after vaccination (MAV), Non-MAV, and control groups*.

GM/KM Markers	MAV % (n=19)	Non-MAV % (n=34)	Control % (n=266)	MAV vs. Non- MAV		MAV vs. Control		Non-MAV vs. Control	
				P-values	OR (95% CI)	P-values	OR (95% CI)	P-values	OR (95% CI)
Allotypes									
GM 2	52.6	23.5	25.9	0.0319	3.61 (1.09-11.99)	0.012	3.17 (1.24-8.13)	0.7642	1.14 (0.49-2.63)
GM 13	94.7	73.5	59.0	0.0756	0.15 (0.02-1.33)	0.0012	12.5 (1.64-95.05)	0.1483	0.52 (0.23-1.15)
KM 1	62.3	23.5	33.3	0.0043	5.57 (1.64-18.94)	0.0087	3.43 (1.30-9.03)	0.3173	1.53 (0.66-3.51)
Phenotypes									
GM 1, 2, 3, 5, 13, 17, 21, 21, 23	26.3	8.8	3.4	0.1181	0.27 (0.06-1.30)	0.001	10.2 (3.01-34.50)	0.1434	0.36 (0.09-1.41)
KM 1, 3	52.6	17.6	25.9	0.0078	5.19 (1.47-18.29)	0.012	3.17 (91.23-8.13)	0.3994	1.63 (0.65-4.11)
KM 3, 3	42.1	76.5	63.9	0.0124	0.22 (0.07-0.75)	0.0984	2.43 (0.95-6.26)	0.2087	0.54 (0.23-1.25)

*Conventions and abbreviations per prior Tables.

The allotypes GM 2 (OR = 3.61, 95% CI = 1.09-11.99, p = 0.0319), KM 1 (OR 5.57, 95% CI = 1.64-18.94, p = 0.004), and the phenotype KM1,3 (OR 5.19, 95% CI = 1.47-18.29, p = 0.0078) were risk factors for MAV compared to Non-MAV. Because the JDM subgroup was more frequent in the non-MAV than MAV groups, we performed a sensitivity analysis with the MAV group that received their last vaccination within three months and selecting a random sample of JDM patients to create a similar proportion of myositis clinical subgroups in the non-MAV group as in the MAV group in the three month window. In this analysis, the MAV group’s GM/KM risk alleles remained unchanged comparing the MAV and non-MAV groups.

## 4 Discussion

Gene-environment interactions appear to play an important role in the development of autoimmune diseases (28). Immunogenetic factors are critical for immune responses to vaccines and have been proposed to modulate risk for the development of vaccine adverse reactions (21). This study suggests possible genetic associations with the development of myositis after vaccinations. HLA alleles have been associated with the development of many autoimmune diseases, including multiple sclerosis, systemic lupus erythematosus, type 1 diabetes mellitus, Sjogren disease and IIM (29–34), as well as possible risk factors for some vaccine adverse events (21).

Our study identified HLA-DQA1*03:03 as a unique risk factor for MAV versus HC, as this allele is not known to be associated with any other IIM groups. This unique risk factor for MAV suggests a different immune response pathway leading to myositis after vaccinations. Interestingly, the known myositis risk factor DRB1*03:01 was present in lower frequency in the MAV group compared to non-MAV group.

The frequency of HLA-DQA1*02:01, a known risk factor in Caucasians for anti-Mi-2 autoantibodies, was significantly higher in patients with MAV, particularly after the Hepatitis B and influenza vaccines, compared to non-MAV, but no association of MAV was seen with anti-Mi-2 autoantibodies. However, HLA-DQA1*02:01 appeared to be a protective factor for the non-MAV group compared to the HC. Although DRB1*07 had previously been described to be associated with myositis in certain racial populations (1), we found this allele to be significantly more frequent in Caucasians with MAV after Hepatitis vaccines compared to the non-MAV group. These findings highlight the complex gene-environment interactions involved in MAV and suggest potential areas for future research and interventions.

The results of comparing both MAV and non-MAV to HC revealed significant associations between specific HLA alleles and risk of myositis, showing further alleles of interest in the immunogenetic profiles of these patients. DRB1*10:01 and DQA1*03:01 were linked to an elevated risk of MAV, indicating a genetic predisposition to myositis following immunization. The protective association with DRB1*02 suggests a reduced likelihood of developing myositis in carriers of this allele, potentially due to its role in modulating immune responses. Previous literature has not elucidated any association of these alleles with myositis, warranting further investigations.

Immunoglobulin genes are important risk and protective factors for many autoimmune diseases, and GM13, KM1 and KM3 allotypes have been described as risk factors for myositis (9, 10). The GM/KM analysis identified GM2 and GM13 as risk factors for MAV compared to HC, but not for the non-MAV group. Similarly, KM1 and the KM1,3 phenotype were also linked to increased MAV risk. These findings suggest that specific GM/KM allotypes may serve as additional non-HLA genetic markers for MAV risk, warranting further research into their potential for personalized risk assessment.

Among the 56 MAV patients, there was a median interval of 2.2 months from vaccination to the first myositis symptom. Previous case reports showed the interval between vaccination and the development of symptoms of myositis ranged from 24 hours to 2 months, which generally aligns with our observations (13, 35–37). It has been postulated that when patients develop myositis after repeated vaccine exposure, it is likely due to an amplified immune response triggered by the repeated doses. While our data showed a delayed onset of myositis symptoms following the influenza vaccine, this contrasts with previous case reports that reported a shorter latency period of less than a month after receiving the influenza vaccine (36, 38). As there was a significantly shorter latency period, with a median of one month from vaccination to first myositis symptom for those developing MAV after Hepatitis B vaccine (p = 0.045), it is possible that a different mechanism of immune activation may be at work in these cases.

Our study has several limitations. First, our cohort was relatively small and was collected before the onset of the COVID-19 pandemic, and as a result, it does not include patients who developed myositis after receiving COVID-19 vaccinations. This a notable limitation, particularly in light of numerous case reports that have been published during and after the pandemic documenting the onset of autoimmune diseases, including myositis and specifically anti-melanoma differentiation-associated protein 5 (MDA5) autoantibody-positive DM following COVID-19 vaccination (5, 39–41). Other recently approved vaccines, including those to rotavirus, human papillomavirus, and herpes zoster were also not included in our study. It is interesting that so many different vaccine antigens might be associated with myositis, which suggests a single mechanistic explanation is not likely, and also raises the question of the role of the various adjuvants used in these many vaccines. However, given the small numbers of cases and variations in adjuvants from vaccine to vaccine, from manufacturer to manufacturer, and over time, it was not possible to carefully evaluate this. Furthermore, our investigation did not include certain recently identified myositis autoantibodies, including anti-MDA5, and did not include the most recent genotyping methods. And some non-MAV cases may have received vaccinations that were not recalled or documented, potentially biasing the comparisons. Nevertheless, our study lays the groundwork for future research on MAV. We hope that future research will build on this foundation, incorporating more recent methods and including all vaccines and phenotypes of myositis to provide a more comprehensive understanding of MAV.

## 5 Conclusion

Our study highlights the complex relationship between vaccinations and the onset of myositis. Our findings are generally consistent with previous studies and reports of MAV, although our data showed a somewhat more delayed onset of myositis symptoms after vaccination, particularly following the influenza vaccine. The novel identification of the HLA-DQA1*03:03 allele as a unique risk factor for MAV and the protective factor of HLA-DRB1*03:01 suggests the role of a genetic predisposition in the MAV group that differs from non-MAV myositis patients. GM/KM associations and other HLA genes were noted among specific vaccines and MAV. These genetic associations could provide insights into the pathogenesis of myositis, suggesting that specific gene-environment interactions may influence the susceptibility of developing MAV. Studies in larger populations exploring greater numbers of deeply clinically, immunologically, and genetically phenotyped subjects, and including all currently available vaccines, are needed to understand possible associations among vaccines and myositis and the genetic risk and protective factors involved. A larger study population would also be instrumental in determining the possible epistatic or interactive effects of HLA, GM, and KM alleles on MAV.

## Data Availability

The raw data supporting the conclusions of this article will be made available by the authors, without undue reservation.
